# Chloasma

**Published:** 1851-01

**Authors:** 


*Chloasma.*—This disease of the skin is also known by the names, Ephelis, Maculæ, Hepaticæ, Pityriasis versicolor Leberflectete, and Liver spots: and generally makes its appearance on some part of the chest or arms, and extends in very irregular patches to other parts of the body, sometimes covering nearly its entire surface. As far as the disease spreads, the skin assumes a dull, yellowish or brown color, sometime varying in tints. There is a very slight elevation of the cuticle in most cases, with a very fine eruption. Occasionally the itching is very annoying, though not at all constant. The patches are often covered with minute scales.

This disease is supposed to exist as a sequel to disease of the stomach or liver; but several cases have certainly come under my observation, where there was no perceptible functional derangement, either of the stomach or liver; and I am of the opinion, therefore, that it has no more connection with derangement of the stomach and liver than has impetigo, lepra or psoriasis.

My principal object in introducing this subject is to speak of the treatment which I think has been heretofore unsatisfactory, both to the physician and to the patient. During the early years of my practice, the cure of this superficial disease annoyed me exceedingly. In 1844, began to use the Sulphur Fume Bath as a remedy, and from that time have had entire success; and am now prepared to recommend this remedy as a specific for this disease, if there be any specific in medicine. In recent cases a few applications are sufficient; and in no case has it been necessary to apply it more than eight or ten times. If any member of the profession has a remedy as certain as this, and more easily applied, it would be highly gratifying to have it made more public.

*Manchester, Nov.* 1850. WILLIAM GREY, M. D.

*New Hampshire Med. Jour.*

